# Attitudes of Slovenian family practice patients toward changing unhealthy lifestyle and the role of family physicians: a cross-sectional study

**DOI:** 10.3325/cmj.2011.52.205

**Published:** 2011-04

**Authors:** Zalika Klemenc-Ketis, Mateja Bulc, Janko Kersnik

**Affiliations:** 1 Department of Family Medicine, Medical School, University of Maribor, Maribor, Slovenia; 2 Department of Family Medicine, Medical School, University of Ljubljana, Ljubljana, Slovenia

## Abstract

**Aim:**

To assess patients’ attitudes toward changing unhealthy lifestyle, confidence in the success, and desired involvement of their family physicians in facilitating this change.

**Methods:**

We conducted a cross-sectional study in 15 family physicians’ practices on a consecutive sample of 472 patients (44.9% men, mean age  [± standard deviation] 49.3 ± 10.9 years) from October 2007 to May 2008. Patients were given a self-administered questionnaire on attitudes toward changing unhealthy diet, increasing physical activity, and reducing body weight. It also included questions on confidence in the success, planning lifestyle changes, and advice from family physicians.

**Results:**

Nearly 20% of patients planned to change their eating habits, increase physical activity, and reach normal body weight. Approximately 30% of patients (more men than women) said that they wanted to receive advice on this issue from their family physicians. Younger patients and patients with higher education were more confident that they could improve their lifestyle. Patients who planned to change their lifestyle and were more confident in the success wanted to receive advice from their family physicians.

**Conclusion:**

Family physicians should regularly ask the patients about the intention of changing their lifestyle and offer them help in carrying out this intention.

Unhealthy lifestyle, including unhealthy diet and physical inactivity, is still a considerable health problem all over the world. Despite publicly available evidence about the health risks of unhealthy lifestyle, people still find it hard to improve their unhealthy diet and increase physical activity. Previous studies have shown that attitudes toward lifestyle change depended on previous health behavior, awareness of unhealthy lifestyle, demographic characteristics, personality traits, social support, family functioning, ongoing contact with health care providers, and an individual’s social ecology or network (1-4).

As community-based health education approaches have had a limited effect on health risk factors reduction (3,5), the readiness-to-change approach, based on two-way communication, has become increasingly used with patients who lead an unhealthy lifestyle (3,6,7). Family physicians are in a unique position to adopt this approach, since almost every patient visits his/hers family physician at least once in five years (8). Previous studies showed that patients highly appreciated their family physicians’ advice on lifestyle changes (9,10). Moreover, patients who received such advice were also more willing to change their unhealthy habits (3,7,11). The reason for this is probably that behavioral changes are made according to the patient’s stage of the motivational circle at the moment of consultation (12), which can be determined only by individual approach.

Although family physicians are convinced that it is their task to give advice on health promotion and disease prevention, in practice they are less likely to do so (13). The factors that prevent them from giving advice are time (14,15), cost, availability, practice capacity (14), lack of knowledge and guidelines, poor counseling skills (16), and personal attitudes (17). It also seems that physicians’ assessment varies considerably according to the risk factor in question. For example, information on diet and physical activity are often inferred from patients’ appearance rather than from clinical measurements (14). Also, health care professionals seldom give advice on recommended aspects of intervention that could facilitate behavioral change (18). As a large proportion of primary care patients are ready to lose weight, improve diet, and increase exercise (19), it is even more important that their family physicians provide timely advice.

So far, several studies have addressed patients’ willingness to make lifestyle change (2-5,20) and the provision of professional advice (3,5,7,10,11). However, none of these studies have investigated the relation between these factors. So, the aim of our study was to assess the relation between patients’ attitudes toward changing unhealthy lifestyle, confidence in success, and the desired involvement of their family physicians in facilitating the change.

## Methods

### Study design

This was a cross-sectional study performed in family practice settings in Slovenia as part of a study (EUROPREVIEW) that was carried out by the European Network for Prevention and Health Promotion in Family Medicine and General Practice (EUROPREV) (unpublished findings). The study was approved by the National Ethics Committee.

### Study population

The sample included family physicians from 15 family practices; 50% from rural or semi-rural and 50% from urban settings. We randomly chose family practices using random seed numbers from the list of 156 Slovenian tutors’ family practices (in year 2007) and none of the selected family physicians refused to participate. Physicians invited 600 consecutive patients between 30 and 70 years old, stratified by sex and age, ie, 40 patients per study practice: 10 men and 10 women 30-49 years old, and 10 men and 10 women 50-70 years old.

### Sample calculation

We aimed at a minimum of 398 participants, considering that the response rate of patients who did not receive reminders for participating in the preventive care varied between 49% and 62% (8,13). Therefore, taking into consideration 0.5 patients who will not respond, the maximum acceptable difference of 0.05, and an α error of 0.05, the required sample size was 378 patients. Assuming a non-participation rate of 5% of selected patients, the sample size was increased to a minimum of 398 patients in order to compensate for the anticipated loss.

### Data collection

Data were collected from October 2007 to May 2008. Before distributing the self-administered anonymous questionnaires, the physician or the practice nurse had explained the study’s protocol and aim to patients and obtained their informed consent.

For the Slovene survey, the original EUROPREV questionnaire was translated from English to Slovene and translated back to English using a standard procedure.

The questionnaire consisted of 4 sections: patients’ demographic data (Table 1) and health history, lifestyle habits (diet, physical activity, smoking, risky alcohol consumption, and overweight), planned changes of unhealthy behavior, and perception of preventive activities conducted by his/her family medical team.

**Table 1:** Demographic characteristics of Slovenian primary health patients included in the study on attitudes toward lifestyle change

Characteristic	No. (%)
Sex (n = 472):	
men	212 (44.9)
women	260 (55.1)
Marital status (n = 472):	
married or living with a partner	354 (75.0)
single	51 (10.8)
separated or divorced	35 (7.4)
widowed	32 (6.8)
Education level (n = 471):	
primary school	93 (19.7)
secondary school	259 (55.0)
university or postgraduate education	119 (25.3)
Employment status (n = 472):	
employed	371 (67.2)
students	1 (0.2)
housewives	7 (1.5)
retired	126 (26.7)
unemployed	21 (4.4)

In this study, we analyzed only the questions on patients’ attitudes toward changing their unhealthy habits and the advice on life-style changes provided by family physicians (Tables 2-4).

**Table 2:** Planning to change unhealthy diet and the need for family physicians’ advice in Slovenian primary health patients

	No. (%) of patients who answered	
Item	yes	no
Do you plan to:		
improve eating habits (n = 472)	95 (20.1)	50 (10.6)
increase physical activity (n = 470)	96 (20.3)	41 (8.7)
reach normal weight (n = 469)	98 (20.8)	41 (8.7)
Would you like to receive advice from your family physician to help you:		
improve eating habits (n = 217)	159 (33.7)	58 (12.3)
increase physical activity (n = 208)	145 (30.7)	63 (13.3)
reach normal weight (n = 203)	139 (29.4)	64 (13.6)

**Table 4:** The relationship between the confidence in successful changing of unhealthy lifestyle habits and the need for family physicians’ advice in Slovenian primary health patients*

Items	Would you like to receive advice from your family physician to to help you: (mean ± standard deviation):								
	improve eating habits			increase physical activity			reach normal weight		
	yes	no	*P*	yes	no	*P*	yes	no	*P*
How confident are you that you can:									
improve eating habits	2.7 ± 0.8	2.3 ± 0.9	0.005	2.7 ± 0.9	2.3 ± 0.9	0.012	2.8 ± 0.9	2.4 ± 0.9	0.050
increase physical activity	2.8 ± 0.8	2.4 ± 0.9	0.009	2.8 ± 0.8	2.4 ± 0.8	0.003	2.9 ± 0.8	2.4 ± 0.8	0.001
reach normal weight	2.8 ± 0.8	2.3 ± 0.8	0.002	2.7 ± 0.9	1.9 ± 0.8	0.004	2.6 ± 0.8	2.4 ± 0.8	0.001

*Scores on a scale from 1 to 4 (from 1 - no confident to 4 - very confident or 1 - not applicable to 4 - yes to advice from family physicians. Statistics: Independent *t* test.

Questions on planning the life-style changes had 6 possible answers: not applicable; no intention of changing in the next 6 months; planning to change during the next 6 months; planning to change during the next month; currently changing; I don’t know.

Questions on confidence in success had answers on a 4-point Likert scale: 1 point – not at all confident; 2 points – doubtful; 3 points – confident; 4 points – very confident.

Questions on receiving professional advice from the family physicians had 4 answers: not applicable; no; I don’t know; yes.

### Statistical analysis

We used SPSS, version 13.0 (SPSS Inc., Chicago, IL, USA) and calculated descriptive statistics. For bivariate analysis, we used independent *t* test, χ^2^-test, and linear correlation. Significance level was set at *P* < 0.05. The variable about life changes planning were dichotomized in the following way: answers “not applicable,” “I don’t know,” and “I’m currently changing” were excluded from the analysis; the answer “no intention of changing in the next 6 months” was coded as “no planned changes;” and the answers “I’m planning to change during the next 6 months” and “I’m planning to change during the next month” were coded as “planned changes.” We also dichotomized the “advice from family physician” variable in the following way: answers “not applicable” and “I don’t know” were excluded from the analysis; the answer “no” was coded as “no need for advice;” and the answer “yes” was coded as “a need for advice.”

## Results

### Demographic characteristics

Out of 472 patients included in the study (response rate 78.7%), 212 (44.9%) were men (Table 1). Mean age ± standard deviation of the sample was 49.3 ± 10.9 years. On average, patients completed 12.3 ± 3.8 years of education (Table 1).

### Planning of lifestyle changes

Nearly 20% of patients planned to change their eating habits, increase physical exercise, and reach normal body weight (Table 2). More women than men planned to reach normal body weight (78.2% vs 60.7%, *P* = 0.039). Patients who planned to change their level of physical activity significantly more frequently planned to change their eating habits and reach normal body weight than those who did not plan to change the level of physical activity (93.8% vs 11.8%, *P* < 0.001; 90.9% vs 18.2%, *P* < 0.001, respectively). Patients who planned to change their eating habits significantly more frequently planned to lose their weight than those who did not plan to change their eating habits (98.5% vs 16.7%, *P* < 0.001).

### Confidence in successful life-style changes

Patients were not very confident that they could improve their eating habits, increase physical activity, or reach normal body weight (2.5 ± 0.9, 2.7 ± 0.8, 2.7 ± 0.8; respectively).

Patients with more completed years of education were more confident that they could improve their eating habits, increase physical activity, or reach normal body weight (r = 0.260, *P* < 0.001; r = 0.201, *P* = 0.002; r = 0.241, *P* < 0.001, respectively). Also, younger patients were more confident that they could make the planned changes (r = -0.192, *P* = 0.004; r = -0.203, 0*P* = 0.002; r = -0.230, *P* < 0.001, respectively).

The confidence that they would improve eating habits was significantly correlated with the confidence that they would increase physical activity (r = 0.683, *P* < 0.001) and reach normal body weight (r = 0.734, *P* < 0.001). The confidence that they would increase physical activity was also significantly correlated with the confidence that they would reach normal body weight (r = 0.770, *P* < 0.001).

### Involvement of family physicians

Approximately 30% of patients said that they would like to receive professional advice from their family physicians to change their eating habits, increase physical activity, and reach normal body weight (Table 2). More men than women said that they would like to receive the advice on how to change their eating habits and reach normal body weight (81.0% vs 66.7%, *P* = 0.021; 78.3% vs 61.7%, *P* = 0.014, respectively).

Patients who planned to change some of their habits or were more confident in success also said that they would like to receive the advice from their family physician (Tables 3 and 4).

**Table 3:** The relationship between the intention of changing unhealthy lifestyle and the need for family physicians’ advice in Slovenian primary health patients

Items	Would you like to receive advice from your family physician to help you: (%)								
	improve eating habits			increase physical activity			reach normal weight		
	yes	no	P*	yes	no	P*	yes	no	P*
Do you plan to:									
improve eating habits	81.7	46.7	0.001	76.8	50.0	0.024	82.0	55.6	0.017
increase physical activity	81.5	66.7	0.224	81.3	55.6	0.018	84.9	65.0	0.101
reach normal weight	78.3	72.0	0.585	74.6	72.0	0.793	82.4	68.8	0.194

*χ^2^ test.

## Discussion

Our study showed that although a quarter of family practice patients planned to change their unhealthy lifestyle, they were not very confident in their success. Also, almost one third of patients wanted to receive family physicians’ advice on this issue.

Other Slovenian studies also addressed some issues related to this topic (8,21-24) and showed that preventive measures were important both to family physicians and patients. This is also in line with some foreign studies (2,3).

Many factors can influence the readiness for lifestyle changes (8). Our study confirmed the findings of other studies (2,3,25-27) that younger and more educated people had a higher motivational readiness to change. Although it is still not fully understood why these two factors, especially education, play a role in motivation (27), one possible reason could be a greater access to health information (eg, through internet use) and a greater ability to evaluate health risks and benefits when making lifestyle choices (27).

Interestingly, more men than women expressed the wish to receive advice from their family physicians on how to change their unhealthy lifestyle. This is somewhat unexpected since it is already known that men express less interest in their health than women and less often seek advice from their family physicians (28). On the other hand, women practice a healthier lifestyle (1) and perhaps do not need such advice. Some previous studies reported that women received less advice than men (10,29), some reported the opposite (6), and some found no effect of sex (7,1).

Patients who planned to change their eating habits wanted to receive advice from their family physician on all three lifestyle factors: diet, physical activity, and body weight. On the other hand, patients who planned to increase their physical activity only wanted advice on this particular risk factor. It seems that patients associated changing their eating habits with increasing physical activity and lowering body weight, but did not associate increasing the physical activity with changing the eating habits and lowering body weight.

Similar to our findings, Taylor et al (5) found that readiness to change and interest in communication with health care providers about the change were significantly related in case of physical activity but not in case of dietary practices. Our findings are encouraging because they indicate greater likelihood for behavioral change in case of eating habits and physical activity. Also, patients are aware of the fact that unhealthy eating habits, physical inactivity, and excessive body weight cannot be managed separately because they are closely related to each other.

Patients who wanted to reach normal body weight did not need their family physicians’ advice. This is an interesting finding, because losing weight is mostly a very difficult process that requires a multidisciplinary approach (30). Also, many weight-loss diets available in public are unhealthy and have a contradictory long-term effect. Wee et al (19) demonstrated that although a large proportion of primary care patients were at advanced stages of readiness to lose weight, physicians were those who initiated the majority of discussions on weight loss (18). This is consistent with our findings about patients’ need for advice on weight loss.

Our finding that patients who want to change lifestyle habits also want to receive advice is consistent with the motivational circle (12): individuals at the stages of contemplation, preparation, or action require more attention. This underlines the fact that family physicians, when motivating patients for lifestyle changes, should adhere to the activity guidelines proposed for each stage of motivational circle. This is also supported by our finding that patients who are more confident in the success significantly more often ask for advice from their family physicians.

Unfortunately, previous studies showed that family physicians were frequently not providers of such advice (31) and often gave their advice to wrong people (32). Family physicians should bear in mind that their patients do think about changing their lifestyle and need help in doing so. They should regularly ask their patients about these intentions and be aware of the patient’s current stage of motivational circle and the appropriate actions to be done at each stage. Special attention should be directed to men, patients with lower education, and older patients as they have less intention of changing habits and less confidence in success, and are therefore at greater cardiovascular risk. Also, when patients ask for advice on healthy diet, family physicians should, if appropriate, also give advice on physical activity and body weight.

The main strengths of our study are a large sample, good response rate that corresponds to sample size calculation, stratified sample, and the inclusion of consecutive patients. There are also some limitations, the most important of which is generalizability of results. Rather than having used a representative sample of family practices, we chose the practices from a list of tutors’ family practices and excluded other family practices in Slovenia. The results also cannot be generalized to other countries. However, since we performed random selection of family practices and stratification according to type of area, and obtained similar results to other studies, we believe that our study is not considerably biased. Furthermore, the study design did not allow us to associate patients’ actual clinical status, life style habits, and motivation stage with their attitudes to change. We also did not assess the lifestyle habits of those patients who were willing to change, so we do not know whether these patients led an unhealthy lifestyle.

Further studies should address patients’ clinical status, actual life style habits, and motivation stage, and connect these features to their attitudes, confidence, and willingness to change. Also, the provision of advice from family physicians should be addressed more extensively. Regardless of the limitations, we believe that our findings could present a basis for the development of guidelines for motivational management of family medicine patients.
